# Comparison of Dosage of Glucocorticoid in Idiopathic Membranous Nephropathy: A Systematic Review and Network Meta-Analysis

**DOI:** 10.7759/cureus.51936

**Published:** 2024-01-09

**Authors:** Yanhua Li, Ziqing Gao, Jianhong Zhu, Jianan Su, Pengwei Chen, Jiande Li, Min Feng

**Affiliations:** 1 Department of Rheumatology, Nanhai District People's Hospital, Foshan, CHN; 2 Department of Nephrology, Sun Yat-sen Memorial Hospital, Guangzhou, CHN; 3 Department of Pharmacy, Sun Yat-sen Memorial Hospital, Guangzhou, CHN; 4 Department of Pain Management, The First People's Hospital of Foshan, Foshan, CHN

**Keywords:** network meta-analysis, systematic review, immunosuppressant, glucocorticoid, idiopathic membranous nephropathy

## Abstract

Purpose: Idiopathic membranous nephropathy (IMN) with moderate risk or above was recommended to receive immunosuppressive therapy. We attempted to evaluate the optimal dose of glucocorticoid when combined with evidence-proven effective immunosuppressants by network meta-analysis.

Methods: A systematic review of the literature was conducted in PubMed, Embase, Cochrane Library, and ClinicalTrials.gov from inception until January 2022. Randomized controlled trials (RCTs) in IMN limited to supportive care, glucocorticoids, cyclophosphamide, chlorambucil, calcineurin inhibitors (CNIs), and rituximab were screened.

Results: Twenty-eight RCTs of 1,830 patients were included. Therapeutic regimens were divided as follows: moderate- to high-dose glucocorticoids plus CNIs (HMSCn), moderate- to high-dose glucocorticoids plus cyclophosphamide (HMSCt), moderate- to high-dose glucocorticoids plus chlorambucil (HMSCh), zero- to low-dose glucocorticoids plus CNIs (LNSCn), zero- to low-dose glucocorticoids plus cyclophosphamide (LNSCt), rituximab alone (R), glucocorticoids alone (SE), and supportive care alone (SP). Compared with SP, HMSCh (risk ratio [RR]: 1.77, 95% confidence interval [CI]: 1, 3.18), HMSCn (RR: 2.5, 95%CI: 1.25, 5.11), HMSCt (RR: 2.15, 95%CI: 1.29, 3.64), LNSCn (RR: 2.16, 95%CI: 1.25, 3.95), and R (RR: 2.07, 95%CI: 1, 4.39) had a higher probability of total remission rate, while HMSCn represented the highest probability depending on the surface under the cumulative ranking area (SUCRA) ranking values. Regarding infection, no significant difference was found between different doses of glucocorticoids plus the same immunosuppressant. HMSCn and HMSCt showed superiority in reducing 24-hour urine total protein compared with HMSCh, LNSCn, SE, and SP, while HMSCn seemed to be the most effective regimen through the ranking of SUCRA value.

Conclusion: Moderate- to high-dose glucocorticoids showed superiority in proteinuria remission when combined with CNIs in IMN, with no increasing risk of infection.

## Introduction and background

Membranous nephropathy (MN) is a kidney-specific autoimmune disease, characterized by the thickening of the glomerular basement membrane due to the deposition of subepithelial immune complexes. About 80% of MN are idiopathic (idiopathic membranous nephropathy, IMN), while the rest 20% are secondary to other diseases or exposures including systematic autoimmune diseases, infection, malignancy, drugs, and so forth [1]. IMN is the most common cause of primary nephrotic syndrome in adults worldwide [2]. Most IMNs are mediated by antibodies to the M-type phospholipase A2 receptor (anti-PLA2R) (85%) and the thrombospondin type 1 domain containing 7A (THSD7A) (3%-5%) [3]. Clinically, 80% of patients with IMN present with nephrotic syndrome and 20% with non-nephrotic range proteinuria. If untreated, about one-third of IMN patients undergo spontaneous remission, one-third progress to end-stage renal disease over 10 years, and the rest remain non-progressive [4].

According to the Kidney Disease Improving Global Outcomes (KDIGO) 2021 clinical practice guideline [5], IMN patients with moderate risk or above were recommended to receive immunosuppressive therapy. Chlorambucil, cyclophosphamide, calcineurin inhibitors (CNIs), and rituximab were proven to show effectiveness in reducing proteinuria in IMN depending on previous studies [6]. Except for immunosuppressive agents, glucocorticoids were recommended as either add-on or not [5]. However, the optimal dosage of glucocorticoids balancing the effectiveness and the adverse events was not recommended in the KDIGO 2021 guideline. Few studies have directly compared the dosage of glucocorticoids in IMN patients.

Therefore, we conducted a systematic review and network meta-analysis of randomized controlled trials (RCTs) to evaluate the efficacy and adverse effects of glucocorticoids with different dosages when combined with recommended immunosuppressants.

## Review

Materials & methods

This network meta-analysis was conducted according to the Preferred Reporting Items for Systematic Review and Meta-analysis (PRISMA) statement [7] and the extension for network meta-analysis [8]. The study was registered prospectively with the International Prospective Register of Systematic Reviews (PROSPERO; CRD42021291150).

Data Sources and Search Strategy

A systematic review of the literature was conducted in PubMed, Embase, Cochrane Library, and ClinicalTrials.gov from inception until January 2022 with relevant records retrieved. RCTs for investigating the treatment of IMN in adults with nephrotic syndrome were targeted, limiting to therapeutic strategies of supportive care, glucocorticoid, cyclophosphamide, chlorambucil, CNIs, and rituximab. Details of the search strategies [9] and results are shown in supplementary table S1.

Study Selection

The inclusion criteria were displayed as follows: 1) RCTs of patients over 14 years old diagnosed as IMN by renal biopsy, excluding secondary membranous nephropathy; 2) therapeutic arms limited to supportive therapy; glucocorticoid alone or combined with immunosuppressive agents (cyclophosphamide, chlorambucil, CNIs including either cyclosporine or tacrolimus, or rituximab); immunosuppressants mentioned above alone; 3) at least one of the following outcomes were mentioned: complete remission (CR) rate, partial remission (PR) rate, change in 24-hour urine total protein (24-hour UTP), relapse rate, and adverse event (infection) rate; 4) literature published in English. Depending on the dosage of glucocorticoid, regimens of immunosuppressive drugs with glucocorticoid were divided into zero- to low-dose glucocorticoid group (prednisone＜5 mg/kg.d^-1^ or equivalent) and moderate- to high-dose glucocorticoid group (prednisone ≥0.5 mg/kg.d^-1^ or equivalent). CR was defined as 24-hour UTP <0.3 g (urine protein: creatinine ratio [uPCR]<300 mg/g or <30 mg/mmol), along with normal serum creatine (SCr). PR was defined as 24-hour UTP <3.5 g (uPCR < 3500 mg/g or <350 mg/mmol) and at least 50% reduction from peak values, accompanied by a stable SCr. Relapse was defined as the development of proteinuria >3.5 g/day after CR or PR [5,10].

Studies were excluded as follows: 1) secondary membranous nephropathy; 2) patients with kidney transplantation; 3) treatments including agents with uncertain outcomes in IMN, such as traditional Chinese medicine, mycophenolate mofetil, mizoribine, leflunomide, azathioprine, and adrenocorticotropic hormone; 4) containing more than one immunosuppressive agent in a single therapeutic arm except for glucocorticoid; 5) comparison of the dosage or administration way of the same immunosuppressant except for glucocorticoid; 6) reviews, meta-analysis, case reports, case series, retrospective cohort studies, single-arm studies, animal studies, and in vitro studies (Table 1).

**Table 1:** Inclusion and exclusion criteria for the systematic review and network meta-analysis IMN, idiopathic membranous nephropathy.

Inclusion criteria	Exclusion criteria
Randomized controlled trials	Reviews, meta-analysis, case reports, case series, retrospective cohort studies, and single-arm studies
Patients over 14 years old with IMN	Secondary membranous nephropathy; patients with kidney transplantation
Therapeutic arms limited to supportive therapy; glucocorticoid alone or combined with immunosuppressants; immunosuppressants alone	Treatments including agents with uncertain outcomes in IMN; more than one immunosuppressant in a single treatment arm; comparison of the dosage or administration way of the same immunosuppressant (except for glucocorticoid)
Studies with defined outcomes	Irrelevant or incomplete outcomes
English studies	Non-English studies

Outcome Assessment

The primary outcome was the total remission rate (TRR), defined as the rate of either CR or PR. The secondary outcomes included adverse event (infection) rate, change in 24-hour UTP, and relapse rate.

Data Extraction and Quality Assessment

Two researchers (YL and ZG) extracted data independently from the included studies. Any disagreement would be resolved by a third reviewer (JZ). The following information was collected: study information (author, year of publication, country, single or multicenter, study type, sample size, intervention, control arm, and follow-up time), characteristics of the patients (age, sex, baseline of 24-hour UTP, and SCr), and primary and secondary outcomes (TRR, infection rate, change in 24-hour UTP, and relapse rate). In studies that reported median and range of 24-hour UTP, the following formula was used to transform it into mean and standard deviation (SD): mean ≈$\left(\frac{4}{4+n^{0.75}}\right)\frac{a+b}{2}+\left(\frac{n^{0.75}}{4+n^{0.75}}\right)m$, and SD ≈ $\frac{b-a}{2{\Phi}^{-1}\left(\frac{n-0.375}{n+0.25}\right)}$ (a for the minimum value; b for the maximum value; m for the median; n for the sample size; 𝛷^-1^ for 𝛷^-1(𝔃)^, the upper 𝔃th percentile of the standard normal distribution) [11,12]. The mean difference (MD) was calculated as the difference of mean values between the follow-up and baseline. The formula of SDChange = $\sqrt{{SD(Baseline)}^2+{SD(Final)}^2-(2\times R\times S D(Baseline)\times S D(Final))}$ was used to calculate the SD of change scores. R represented the Pearson correlation coefficient, inputted with 0.5 [13]. Quality assessment of the included studies was evaluated independently by two researchers (YL and ZG) under the indications recommended in the Cochrane Handbook [14].

Statistical Analysis

Conventional pairwise meta-analysis was initially performed considering the available head-to-head comparisons with a standard random-effect model. Categorical data was estimated by risk ratio (RR) with 95% confidence interval (CI), while continuous variable was evaluated with MD with 95%CI. Between-study heterogeneity was assessed by I^2^ statistic, when I^2^ <50% represented no significant heterogeneity.

A random-effect network meta-analysis was performed by a Bayesian network framework with a Monte Carlo Markov chain (MCMC) model [15]. Four chains were set simultaneously, and the number of tuning iterations and simulation iterations were 20,000 and 50,000, respectively. For all outcomes, evidence from the included studies was summarized in network relation graphs. Outcomes of different therapeutic regimens were ranked through the surface under the cumulative ranking area (SUCRA) curve and demonstrated in league tables. Larger SUCRA scores indicated greater probability [16]. A global heterogeneity was assessed by I^2 ^statistic. To evaluate the consistency between direct and indirect comparisons, node node-splitting approach was performed. A p-value <0.05 was considered statistically significant [17]. All analyses were conducted using R V4.1.2 (R Foundation for Statistical Computing, Vienna, Austria) (pairwise meta-analysis, network meta-analysis, league tables, and assessment of heterogeneity and consistency) and STATA V15.1 (StataCorp LLC, College Station, TX) (network plots and SUCRA curve).

Results

Study Selection and Characteristics

**Figure 1:**
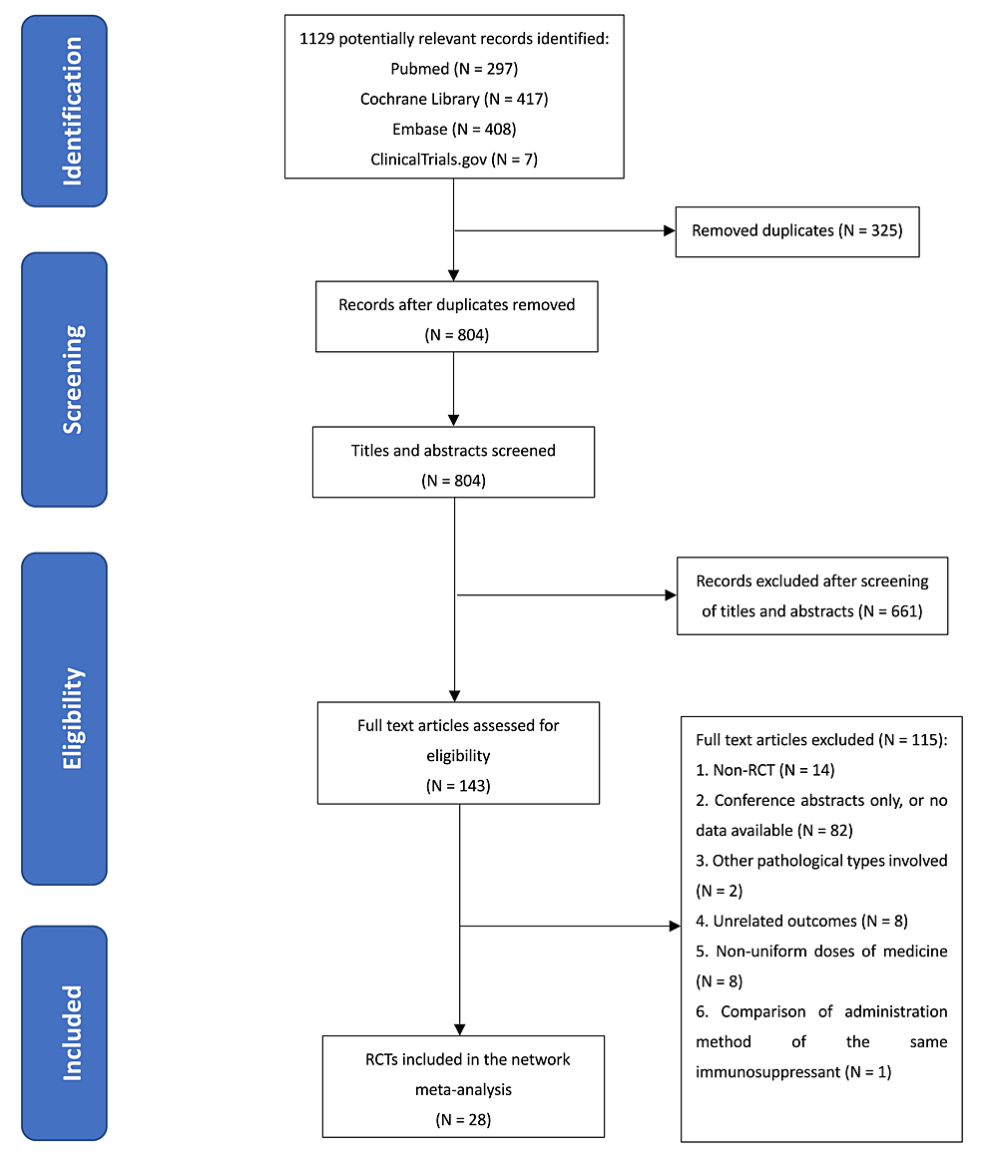
Flowchart of the study selection procedure. RCT, randomized clinical trial.

A total of 1,129 studies were retrieved after the initial search. After removing 325 duplicate studies, 661 studies were excluded from the screening of titles and abstracts. Then after full text was assessed, 115 were removed. Ultimately, 28 RCTs of 1,830 patients were included in the network meta-analysis (Figure 1). Depending on the dosage of glucocorticoids and the type of immunosuppressive agents, therapeutic regimens were divided as follows: a moderate to high dose of glucocorticoids plus CNIs (HMSCn), a moderate to high dose of glucocorticoids plus cyclophosphamide (HMSCt), a moderate to high dose of glucocorticoids plus chlorambucil (HMSCh), zero to a low dose of glucocorticoids plus CNIs (LNSCn), zero to a low dose of glucocorticoids plus cyclophosphamide (LNSCt), rituximab alone (R), glucocorticoids alone (SE), and supportive care alone (SP). Supportive care included non-immunosuppressive antiproteinuric treatment, anticoagulation, and so on. Both supportive care had been administrated or not were eligible in all the immunosuppressive regimens. The main characteristics of the 28 RCTs are summarized in Table 2.

**Table 2:** Characteristics of the eligible trials in the network meta-analysis HMSCh, moderate- to high-dose glucocorticoid plus chlorambucil; HMSCn, moderate- to high-dose glucocorticoid plus calcineurin inhibitors; HMSCt, moderate- to high-dose glucocorticoid plus cyclophosphamide; LNSCn, zero to low-dose glucocorticoid plus calcineurin inhibitors; LNSCt, zero to low-dose glucocorticoid plus cyclophosphamide; R, rituximab alone; SE, glucocorticoid alone; SP, supportive care alone; SCr, serum creatinine; 24-hour UTP, 24-hour urine total protein; SD, standard deviation; IQR, interquartile range; NR, not reported. ^a^Indicates that the unit is mg/dl; bIndicates that the unit is umol/L.

Trial	Location	Single-/multicenter	Intervention	Age (years), mean±SD/ median (range)	Sample size (N)	Number of patients (N)	Baseline 24 h UTP (g/d), mean±SD/ median (IQR/range)	Baseline SCr (mg/dl or umol/L), mean±SD/ median (IQR/range)	Follow-up (months), mean±SD/ median (IQR/range)	Outcome
Jha 2007 [s1]	India	NR	HMSCt	38.00 ± 13.60	93	47	6.11 ± 2.50	1.21 ± 0.31^a^	121 (126-144)	Remission rate, relapse rate, adverse events rate
			SP	37.20 ± 12.40		46	5.91 ± 2.20	1.17 ± 0.22 ^a^		
Ponticelli 1998 [s2]	Italy	Multicenter	HMSCh	50 (18-65)	87	44	7.96 ± 5.19	1.06 ± 0.27^a^	36 (12-78)	Remission rate, relapse rate, adverse events rate
			HMSCt	48 (17-55)		43	6.85 ± 3.51	1.04 ± 0.27^a^	42 (12-72)	
Ponticelli 1989 [s3]	Italy	Multicenter	HMSCh	43.5 (15-70)	81	42	6.18 ± 2.98	93.80 ± 21.50^b^	5 (24-132)	Remission rate, adverse events rate
			SP	42 (16-74)		39	5.30 ± 2.84	93.10 ± 25.30^b^		
He 2013 [s4]	China	Single center	HMSCn	45.40 ± 11.50	56	28	6.76 ± 2.33	81.56 ± 27.22^b^	12	Remission rate, change in 24-hour UTP, relapse rate, adverse events rate
			HMSCt	47.20 ± 13.40		28	6.38 ± 2.19	82.45 ± 26.36^b^		
Ramachandran 2016 [s5]	India	Single center	HMSCt	40.80 ± 10.64	70	35	5.44 ± 2.66	0.91 ± 0.26^a^	12	Remission rate, change in 24-hour UTP, adverse events rate
			HMSCn	38.66 ± 1.91		35	6.67 ± 3.59	0.90 ± 0.27^a^		
Liang 2017 [s6]	China	Single center	HMSCt	53.90 ± 10.40	58	28	6.90 ± 2.20	81.00 ± 22.50^b^	10.5 (0.3-19)	Remission rate, change in 24-hour UTP, relapse rate, adverse events rate
			LNSCn	48.20 ± 13.50		30	5.90 ± 2.70	70.70 ± 17.50^b^	10 (0.2-18)	
Peng 2015 [s7]	China	Single center	HMSCn	43.90 ± 13.20	60	30	11.70 ± 3.20	82.40 ± 13.60^b^	9	Remission rate, change in 24-hour UTP
			HMSCt	40.80 ± 13.30		30	11.90 ± 1.50	78.40 ± 13.80^b^		Relapse rate, adverse events rate
Ponticelli 1992 [s8]	Italy	Multicenter	HMSCh	46 (14-65)	92	45	7.60 ± 4.20	1.00 ± 0.30^a^	54 ± 16	Remission rate, change in 24-hour UTP, adverse events rate
			SE	47 (14-64)		47	7.00 ± 4.10	1.00 ± 0.30^a^	54 ± 17	
Cattran 2001 [s9]	Canada	Multicenter	LNSCn	47.00 ± 11.00	51	28	9.70 ± 5.30	1.30 ± 0.50^a^	19.5	Remission rate, relapse rate
			SE	49.00 ± 14.00		23	8.80 ± 4.70	1.10 ± 0.30^a^		
Chen 2010 [s10]	China	Multicenter	HMSCn	47.20 ± 11.90	73	39	7.71 ± 3.93	75.70 ± 22.40^b^	12	Remission rate, change in 24-hour UTP, relapse rate, adverse events rate
			HMSCt	48.60 ± 11.60		34	7.28 ± 3.91	85.00 ± 37.50^b^		
Cattran 1989 [s11]	Canada	Multicenter	SE	46 (18-77)	158	81	6.90 ± 0.80	120.00 ± 10.00^b^	48.00 ± 3.20	Remission rate, relapse rate
			SP	45 (16-83)		77	5.20 ± 0.90	103.00 ± 9.00^b^		
Reichert 1994 [s12]	Netherlands	Multicenter	HMSCh	45 (31-65)	18	9	8.50 ± 2.50	260.00 ± 112.00^b^	15 (6-36)	Remission rate, adverse events rate
			HMSCt	49 (24-65)		9	9.80 ± 4.80	218.00 ± 85.00^b^		
Li 2015 [s13]	China	Single center	LNSCn	75.10 ± 8.20	27	14	7.20 ± 3.40	98.80 ± 15.10^b^	6-48	Remission rate, adverse events rate
			HMSCn	74.80 ± 7.90		13	7.50 ± 3.80	91.60 ± 20.90^b^		
Kosmadakis 2010 [s14]	Greece	Single center	LNSCn	50.50 ± 4.90	28	10	6.60 ± 1.00	NR	9	Remission rate, change in 24-hour UTP, adverse events rate
			HMSCt	55.40 ± 2.80		8	7.00 ± 0.70	NR		
			SP	51.80 ± 5.40		10	5.20 ± 0.80	NR		
Branten 1998 [s15]	Netherlands	Single center	HMSCh	51.00 ± 12.00	32	15	9.00 ± 2.60	219.00 ± 73.00^b^	38 (8-71)	Remission, change in 24--hour UTP, adverse events rate
			HMSCt	53.00 ± 14.00		17	11.00 ± 5.30	274.00 ± 126.00^b^	26 (5-68)	
Coggins 1979 [s16]	America	Multicenter	SE	16-65	72	34	9.40 ± 6.00	1.10 ± 0.20^a^	23 (4-52)	Remission rate
			SP	16-65		38	8.30 ± 4.00	1.00 ± 0.20^a^		
Fervenza 2019 [s17]	America	Multicenter	R	51.90 ± 12.60	130	65	8.90 (6.80-12.30)	1.30 ± 0.40^a^	24	Remission rate, adverse events rate
			LNSCn	52.20 ± 12.40		65	8.90 (6.70-12.90)	1.30 ± 0.40^a^		
Dahan 2017 [s18]	France	Multicenter	R	53 (42-63)	75	37	NR	98.10 (73.40-122.90)^b^	17 (12.5-24)	Remission rate, adverse events rate
			SP	58.5 (43-64)		38	NR	91.10 (74.30-122.00)^b^	17 (13-23)	
Praga 2007 [s19]	Spain	Multicenter	LNSCn	43.70 ± 12.10	48	25	7.20 ± 3.30	0.98 ± 0.20^a^	18	Remission rate, adverse events rate
			SP	50.10 ± 12.20		23	8.40 ± 5.40	1.10 ± 0.30^a^		
Murphy 1992 [s20]	Australia	NR	LNSCt	47 (26-66)	40	19	5.00 (0.90-13.00)	110.00 (50.00-280.00)^b^	24	Remission rate
			SP	40 (18-65)		21	3.90 (0.50-12.00)	90.00 (50.00-200.00)^b^		
Cattran 1995 [s21]	Canada	Multicenter	LNSCn	44 (22-59)	17	9	11.50 (9.00-18.00)	186.00 ± 65.00^b^	21	Remission rate, change in 24-hour UTP, adverse events rate
			SP	40 (20-61)		8	12.80 (4.00-21.00)	204.00 ± 81.00^b^		
Pahari 1993 [s22]	India	NR	HMSCt	NR	71	36	NR	NR	46 ± 10.2	Remission rate, relapse rate
			SE	NR		35	NR	NR		
Donadio 1974 [s23]	America	NR	LNSCt	25-74	22	11	7.80 (2.00-16.60)	1.20 (0.80-1.90)^a^	12	Remission rate, change in 24-hour UTP, adverse events rate
			SP	26-69		11	7.60 (2.00-12.10)	1.10 (0.80-2.20)^a^		
Scolari 2021 [s24]	Europe	Multicenter	R	54.00 ± 14.00	74	37	6.00 (4.00-10.00)	1.00 ± 0.00^a^	36	Remission rate, relapse rate, adverse events rate
			HMSCt	54.00 ± 17.00		37	6.00 (5.00-9.00)	1.00 ± 0.00 ^a^		
Ponticelli 1984 [s25]	Italy	Multicenter	HMSCh	42.60 ± 10.10	62	32	NR	1.06 ± 0.25^a^	31.4 ± 18.2	Remission rate
			SP	44.90 ± 16.50		30	NR	1.08 ± 0.30^a^	37.0 ± 22.0	
Howman 2013 [s26]	The UK	Multicenter	HMSCh	58.00 ± 12.00	106	33	10.10 ± 5.30	NR	36	Adverse events rate
			LNSCn	58.00 ± 11.00		36	6.80 ± 4.70	NR		
			SP	56.00 ± 16.00		37	9.10 ± 5.30	NR		
Falk 1992 [s27]	America	Multicenter	SE	46.00 ± 13.70	26	13	11.10 ± 6.70	2.70 ± 1.60^a^	29.2 ± 17.1	Change in 24-hour UTP
			HMSCt	43.30 ± 14.80		13	12.40 ± 9.90	2.30 ± 1.00^a^		
Cameron 1990 [s28]	The UK	NR	SE	NR	103	52	10.80 ± 5.90	114.00 ± 42.00^b^	36	Change in 24-hour UTP
			SP	NR		51				

Risk-of-Bias Assessment

According to the Cochrane instrument, more than half of the included studies were judged to have a low risk of bias for random sequence generation. Most of the studies were regarded as low risk of bias for incomplete outcome data and selective reporting, and all studies were at low risk of bias for blinding of outcome assessment. Three studies that used blinding methods were judged to have a low risk of bias. More details of quality assessment are demonstrated in supplementary figure S1.

Heterogeneity Analysis and Evaluation of Inconsistency

For heterogeneity analysis, the global I2 value was 46.56% (TRR), 61.27% (infection rate), 39.28% (change in 24-hour UTP), and 0.00% (relapse rate), respectively. With regard to the local inconsistency, the node-split model revealed no significant discrepancy between the direct and indirect comparisons in outcomes of relapse rate. However, a significant difference was found in one comparison of TRR, one comparison of infection rate, and two comparisons of change in 24-hour UTP (supplementary tables S2-S5).

Conventional Pairwise Meta-Analysis

By conventional pairwise meta-analysis, it was discovered that TRR in groups of HMSCh (RR: 1.37, 95%CI: 1.01, 1.85), HMSCt (RR: 1.46, 95%CI: 1.11, 1.92), and LNSCn (RR: 3.45, 95%CI: 1.54, 7.71) were higher than SE. Besides, the TRR of HMSCh (RR: 2.42, 95%CI: 1.68, 3.48), LNSCt (RR: 2.32, 95%CI: 1.01, 5.32), and R (RR: 1.90, 95%CI: 1.15, 3.13) groups were higher when compared with SP. In terms of infection rate, HMSCt group showed higher probability when compared with LNSCn (RR: 7.23, 95%CI: 1.35, 38.71) and R (RR: 2.50, 95%CI: 1.09, 5.73), respectively. With regard to the outcome of change in 24-hour UTP, HMSCn seemed more effective than HMSCt (RR: -1.19, 95%CI: -1.82, -0.55). HMSCt showed superiority in reducing 24-hour UTP when compared with HMSCh (RR: -6.70, 95%CI: -9.62, -3.78), LNSCn (RR: -1.78, 95%CI: -2.38, -1.18), and SP (RR: -2.80, 95%CI: -3.41, -2.19). In addition, LNSCn could significantly reduce 24-hour UTP when compared with SP (RR: -1.03, 95%CI: -1.72, -0.34). Details of results for pairwise meta-analysis are shown in supplementary tables S6-S9.

Network Meta-Analysis

**Figure 2:**
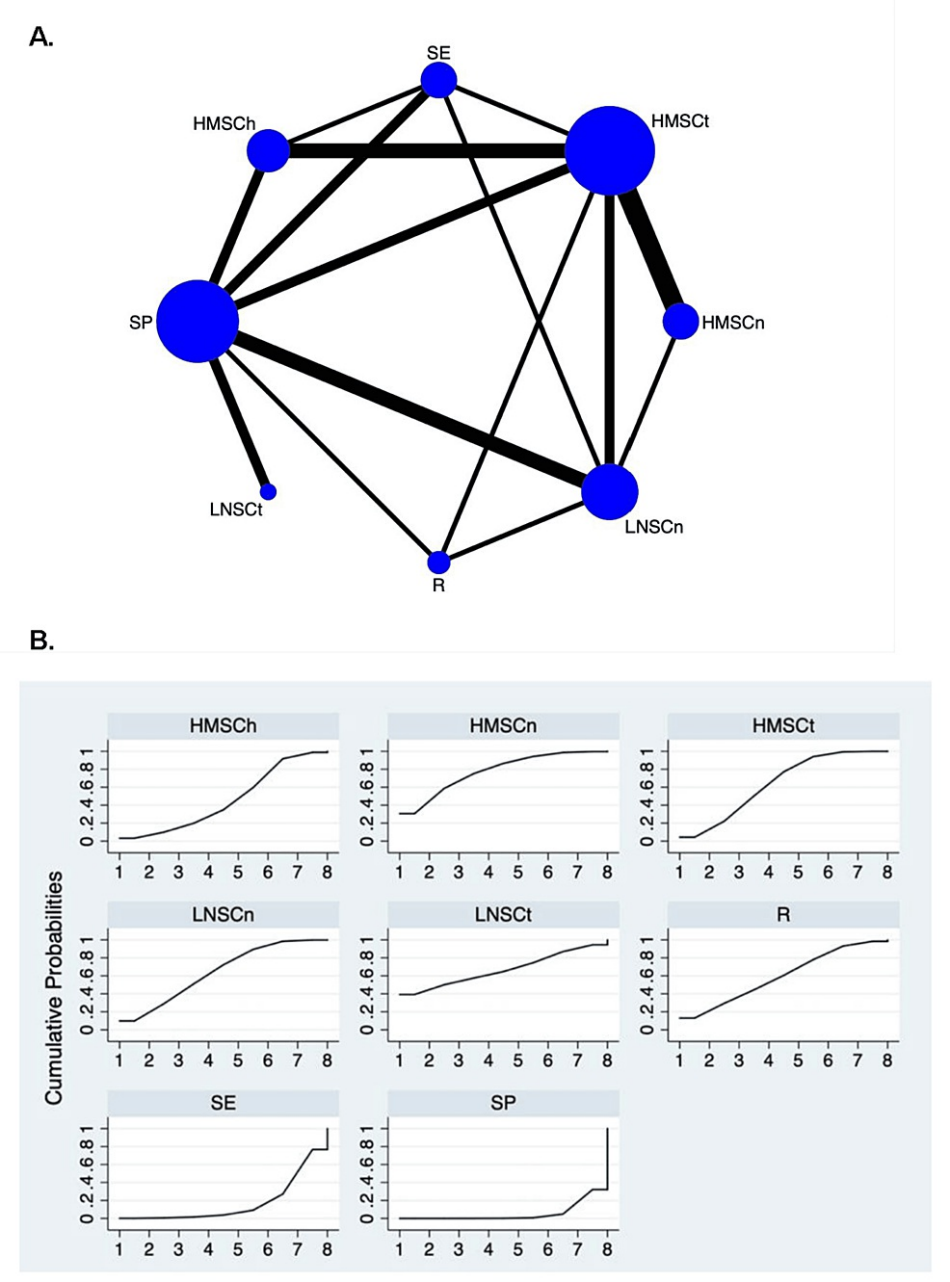
Network map and SUCRA plot for total remission rate in IMN. (A) Network map. Nodes indicate the treatment regimens that are evaluated in the study. The size of the node is proportional to the number of trials evaluating the treatment. Lines represent head-to-head comparisons of the two treatment regimens indicated by the connected nodes. The thickness of the lines is weighted according to the number of trials comparing the two connected treatment regimens. (B) SUCRA plot. SUCRA, surface under the cumulative ranking area; IMN, idiopathic membranous nephropathy; HMSCh, moderate- to high-dose glucocorticoid plus chlorambucil; HMSCn, moderate- to high-dose glucocorticoid plus calcineurin inhibitors; HMSCt, moderate- to high-dose glucocorticoid plus cyclophosphamide; LNSCn, zero to low-dose glucocorticoid plus calcineurin inhibitors; R, rituximab alone; SE, glucocorticoid alone; SP, supportive care alone.

TRR: A total of 25 studies reported that 931 in 1,595 patients achieved remission (CR or PR). The related therapeutic regimens were HMSCh (six trials, 134 patients with remission), HMSCn (five trials, 116 patients with remission), HMSCt (12 trials, 272 patients with remission), LNSCn (seven trials, 117 patients with remission), LNSCt (two trials, 13 patients with remission), R (three trials, 86 patients with remission), SE (five trials, 91 patients with remission), and SP (11 trials, 102 patients with remission). The network map is displayed in Figure 2A. Compared with SP, HMSCh, HMSCn, HMSCt, LNSCn, and R all had a higher probability of TRR (Table 3). With regard to the SUCRA ranking curves, HMSCn seemed to have the highest probability of TRR (Table 4, Figure 2B).

**Table 3:** The league table of all comparisons of total remission rate and adverse event (infection) rate The league table of all comparisons of total remission rate (on the lower triangle) and adverse event (infection) rate (on the upper triangle). The summary estimates are risk ratios (RRs) and 95% confidence intervals. For total remission rate, the column-defining treatment is compared to the row-defining treatment, and RRs >1 favor the column-defining treatment. For adverse event (infection) rate, the column-defining treatment is compared to the row-defining treatment, and RRs <1 favor the row-defining treatment. Significant results are in bold. HMSCh, moderate- to high-dose glucocorticoid plus chlorambucil; HMSCn, moderate- to high-dose glucocorticoid plus calcineurin inhibitors; HMSCt, moderate- to high-dose glucocorticoid plus cyclophosphamide; LNSCn, zero to low-dose glucocorticoid plus calcineurin inhibitors; LNSCt, zero to low-dose glucocorticoid plus cyclophosphamide; R, rituximab alone; SE, glucocorticoid alone; SP, supportive care alone.

Adverse event (infection) rate versus total remission rate							
								HMSCh	0.41 (0.03, 3.31)	0.32 (0.04, 1.53)	0.16 (0.01, 1.01)	0.14 (0.01, 1.48)	0 (0, 0.25)	0.25 (0.02, 1.6)	0 (0, 1037298797754428)
0.71 (0.33, 1.49)	HMSCn	0.77 (0.17, 3.44)	0.39 (0.04, 2.77)	0.35 (0.03, 4.16)	0 (0, 0.8)	0.62 (0.06, 5.32)	0 (0, 2720698666502153)
0.82 (0.47, 1.44)	1.16 (0.7, 1.95)	HMSCt	0.51 (0.08, 2.48)	0.45 (0.05, 3.71)	0 (0, 0.96)	0.8 (0.12, 4.51)	0 (0, 3448403825946490)
0.82 (0.4, 1.61)	1.16 (0.58, 2.25)	1 (0.57, 1.67)	LNSCn	0.88 (0.12, 8.43)	0 (0, 2.1)	1.56 (0.29, 9.89)	0 (0, 7118920290616562)
0.86 (0.37, 1.98)	1.21 (0.51, 2.85)	1.04 (0.5, 2.12)	1.04 (0.51, 2.2)	R	0 (0, 2.53)	1.77 (0.17, 17.24)	0 (0, 7340961391375712)
1.46 (0.76, 2.89)	2.07 (0.96, 4.61)	1.78 (0.97, 3.36)	1.78 (0.94, 3.65)	1.71 (0.74, 4.12)	SE	4827033817.19 (0.77, 8.60129084446801e+33)	19010.61 (0, 5.21995378675354e+34)
1.77 (1, 3.18)	2.5 (1.25, 5.11)	2.15 (1.29, 3.64)	2.16 (1.25, 3.95)	2.07 (1, 4.39)	1.21 (0.66, 2.18)	SP	0 (0, 4160239021124826)
0.74 (0.19, 2.63)	1.04 (0.25, 4.01)	0.9 (0.23, 3.12)	0.9 (0.23, 3.27)	0.86 (0.21, 3.37)	0.5 (0.13, 1.79)	0.42 (0.12, 1.29)	LNSCt

**Table 4:** SUCRA value of total remission rate SUCRA, surface under the cumulative ranking area; HMSCh, moderate- to high-dose glucocorticoid plus chlorambucil; HMSCn, moderate- to high-dose glucocorticoid plus calcineurin inhibitors; HMSCt, moderate- to high-dose glucocorticoid plus cyclophosphamide; LNSCn, zero to low-dose glucocorticoid plus calcineurin inhibitors; R, rituximab alone; SE, glucocorticoid alone; SP, supportive care alone.

Treatment	SUCRA	PrBest	Mean rank
HMSCh	45.4	3.2	4.8
HMSCn	77.6	30.5	2.6
HMSCt	63.9	4.3	3.5
LNSCn	64.3	9.7	3.5
LNSCt	66.8	39.2	3.3
R	59.6	13	3.8
SE	17	0.2	6.8
SP	5.4	0	7.6

Adverse Event (Infection) Rate

**Figure 3:**
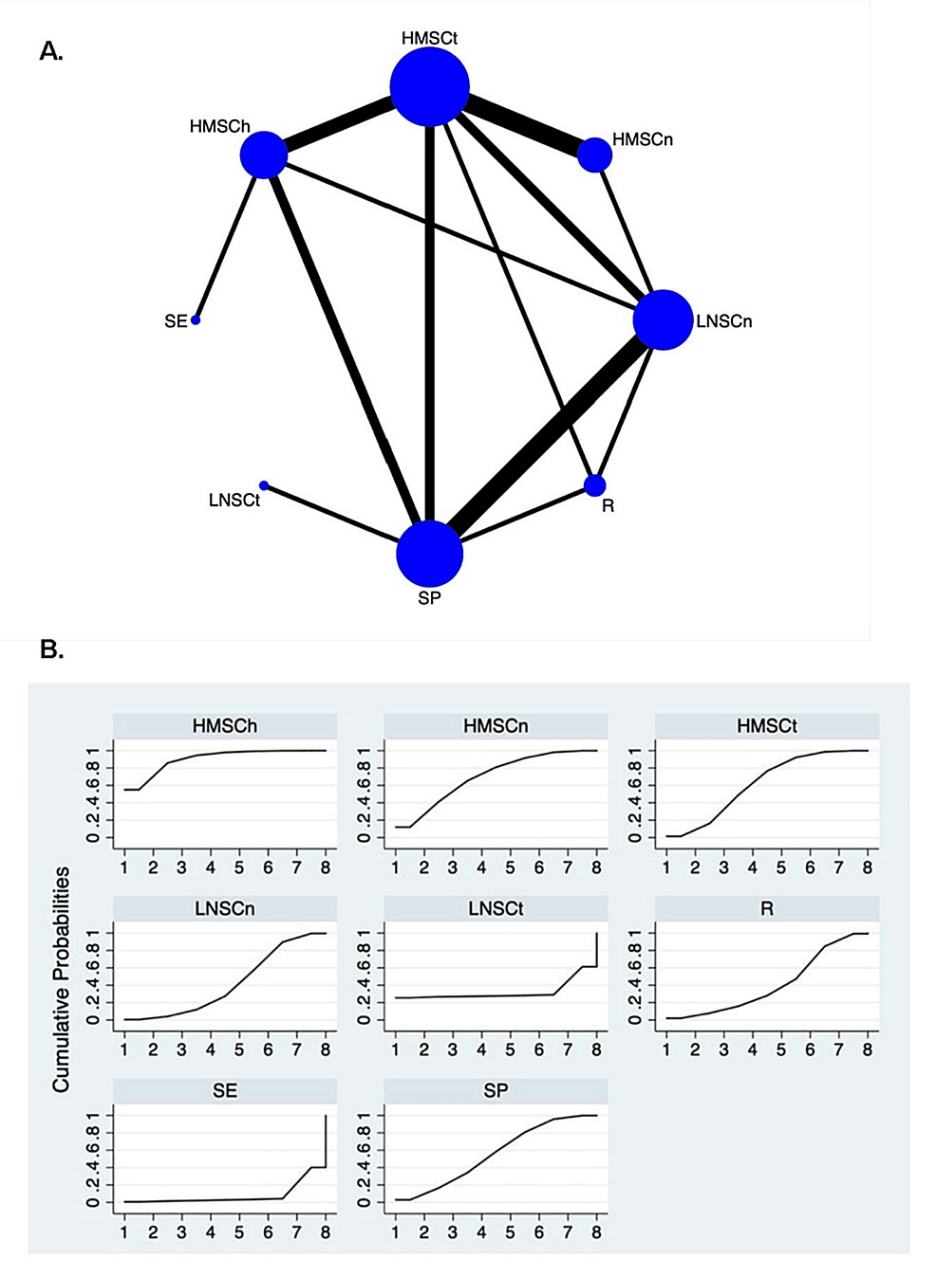
Network map and SUCRA plot for infection rate in IMN. (A) Network map. Nodes indicate the treatment regimens that are evaluated in the study. The size of the node is proportional to the number of trials evaluating the treatment. Lines represent head-to-head comparisons of the two treatment regimens indicated by the connected nodes. The thickness of the lines is weighted according to the number of trials comparing the two connected treatment regimens. (B) SUCRA plot. SUCRA, surface under the cumulative ranking area; IMN, idiopathic membranous nephropathy; HMSCh, moderate- to high-dose glucocorticoid plus chlorambucil; HMSCn, moderate- to high-dose glucocorticoid plus calcineurin inhibitors; HMSCt, moderate- to high-dose glucocorticoid plus cyclophosphamide; LNSCn, zero to low-dose glucocorticoid plus calcineurin inhibitors; R, rituximab alone; SE, glucocorticoid alone; SP, supportive care alone.

Various adverse events were mentioned in the 28 RCTs, such as gastrointestinal symptoms, glucose intolerance, diabetes mellitus, anemia, hypertension, and so forth. However, we mainly focused on infections (pneumonia, urinary tract infection, skin infection, etc.). A total of 204 records of infection in 1,247 patients were mentioned in 20 RCTs, with eight intervention regimens included: HMSCh (six trials, 23 patients with infections), HMSCn (five trials, 35 patients with infections), HMSCt (11 trials, 67 patients with infections), LNSCn (seven trials, 33 patients with infections), LNSCt (one trial, 0 patient with infections), R (three trials, 25 patients with infections), SE (one trial, 0 patient with infections), SP (eight trials, 21 patients with infections). The network plot is demonstrated in Figure 3A. SE was associated with a lower probability of infections compared with HMSCh, HMSCn, and HMSCt when other comparations showed no significant differences (Table 3). Details of SUCRA ranking are displayed in Table 5 and Figure 3B.

**Table 5:** SUCRA value of adverse event (infection) rate SUCRA, surface under the cumulative ranking area; HMSCh, moderate- to high-dose glucocorticoid plus chlorambucil; HMSCn, moderate- to high-dose glucocorticoid plus calcineurin inhibitors; HMSCt, moderate- to high-dose glucocorticoid plus cyclophosphamide; LNSCn, zero to low-dose glucocorticoid plus calcineurin inhibitors; LNSCt, zero to low-dose glucocorticoids plus cyclophosphamide; R, rituximab alone; SE, glucocorticoid alone; SP, supportive care alone.

Treatment	SUCRA	PrBest	Mean rank
HMSCh	90.4	55	1.7
HMSCn	69.9	11.9	3.1
HMSCt	62	1.5	3.7
LNSCn	41.5	0.5	5.1
LNSCt	32.2	25.5	5.7
R	40.7	2	5.2
SE	7.8	0.6	7.5
SP	55.5	2.9	4.1

Change in 24-Hour UTP

**Figure 4:**
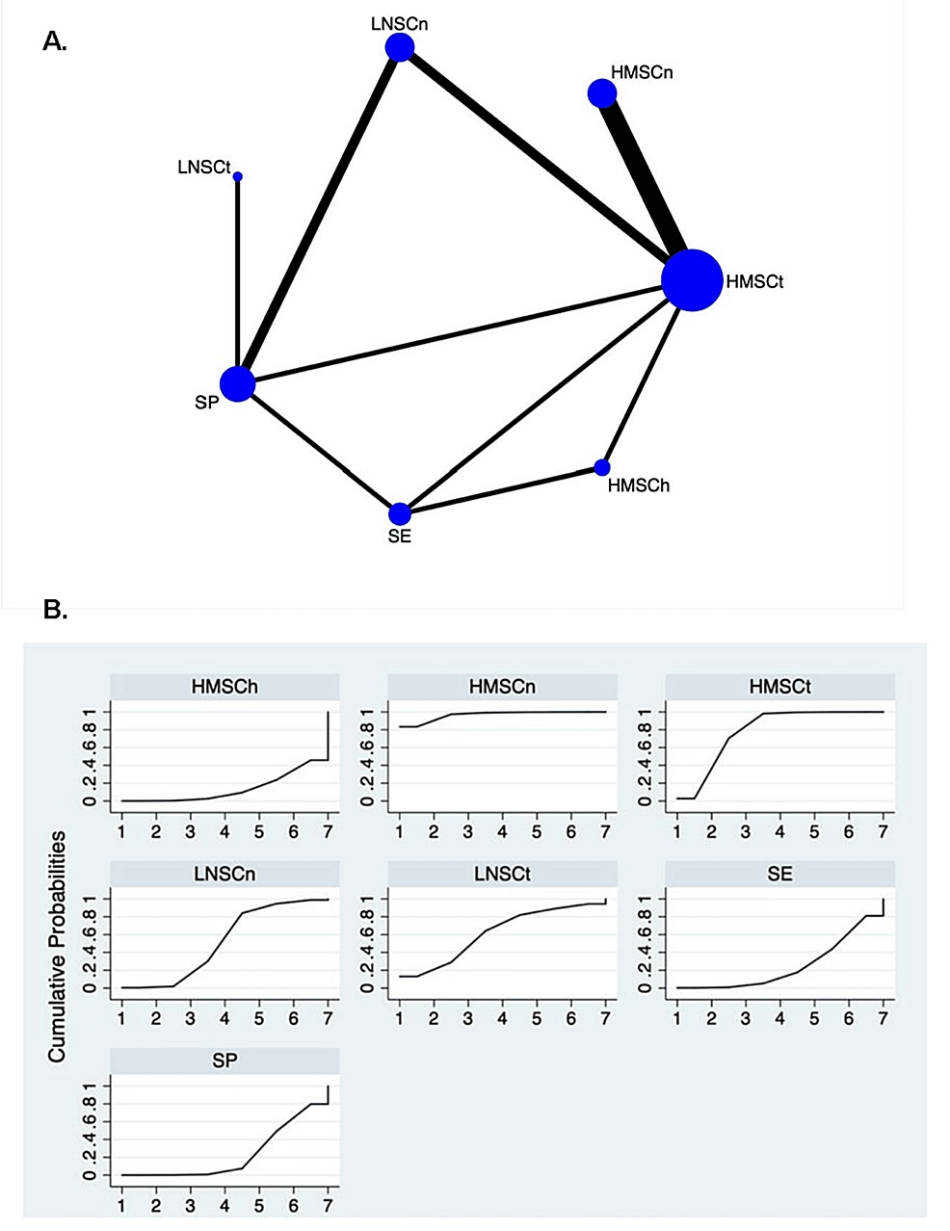
Network map and SUCRA plot for change in 24-hour UTP in IMN. (A) Network map. Nodes indicate the treatment regimens that are evaluated in the study. The size of the node is proportional to the number of trials evaluating the treatment. Lines represent head-to-head comparisons of the two treatment regimens indicated by the connected nodes. The thickness of the lines is weighted according to the number of trials comparing the two connected treatment regimens. (B) SUCRA plot. SUCRA, surface under the cumulative ranking area; 24-hour UTP, 24-hour urine total protein; IMN, idiopathic membranous nephropathy; HMSCh, moderate- to high-dose glucocorticoid plus chlorambucil; HMSCn, moderate- to high-dose glucocorticoid plus calcineurin inhibitors; HMSCt, moderate- to high-dose glucocorticoid plus cyclophosphamide; LNSCn, zero to low-dose glucocorticoid plus calcineurin inhibitors; R, rituximab alone; SE, glucocorticoid alone; SP, supportive care alone.

Twelve RCTs with 595 patients were included in the network meta-analysis. Seven therapeutic regimens were involved: HMSCh (two trials, 41 patients), HMSCn (four trials, 132 patients), HMSCt (eight trials, 193 patients), LNSCn (three trials, 49 patients), LNSCt (one trial, 11 patients), SE (three trials, 89 patients), and SP (four trials, 80 patients). The network map is displayed in Figure 4A. HMSCn and HMSCt showed superiority in reducing 24-hour UTP when compared with HMSCh, LNSCn, SE, and SP (Table 6). By ranking the SUCRA value, HMSCn seemed to be the most effective regimen in reducing 24-hour UTP (Table 7 and Figure 4B).

**Table 6:** The league table of all comparisons of change in 24-hour UTP The summary estimates are mean differences (MDs) and 95% confidence intervals. The column-defining treatment is compared to the row-defining treatment, and MDs <0 favor the column-defining treatment. Significant results are in bold. 24-hour UTP, 24-hour urine total protein; HMSCh, moderate- to high-dose glucocorticoid plus chlorambucil; HMSCn, moderate- to high-dose glucocorticoid plus calcineurin inhibitors; HMSCt, moderate- to high-dose glucocorticoid plus cyclophosphamide; LNSCn, zero to low-dose glucocorticoid plus calcineurin inhibitors; LNSCt, zero to low-dose glucocorticoid plus cyclophosphamide; SE, glucocorticoid alone; SP, supportive care alone.

HMSCh						
4.91 (1.95, 8.17)	HMSCn					
3.79 (1.16, 6.78)	-1.13 (-2.43, 0.29)	HMSCt				
1.97 (-0.96, 5.36)	-2.95 (-5.08, -0.61)	-1.82 (-3.55, -0.01)	LNSCn			
2.76 (-1.92, 7.45)	-2.13 (-6.63, 2.02)	-1.03 (-5.34, 2.88)	0.79 (-3.53, 4.71)	LNSCt		
0.58 (-1.81, 3.46)	-4.33 (-7.16, -1.27)	-3.2 (-5.76, -0.54)	-1.38 (-4.22, 1.5)	-2.18 (-6.39, 2.4)	SE	
0.64 (-2.36, 3.69)	-4.23 (-6.87, -2.03)	-3.1 (-5.38, -1.36)	-1.28 (-3.63, 0.46)	-2.11 (-5.7, 1.5)	0.05 (-2.69, 2.36)	SP

**Table 7:** SUCRA value of change in 24-hour UTP SUCRA, surface under the cumulative ranking area; 24-hour UTP, 24-hour urine total protein; HMSCh, moderate- to high-dose glucocorticoid plus chlorambucil; HMSCn, moderate- to high-dose glucocorticoid plus calcineurin inhibitors; HMSCt, moderate- to high-dose glucocorticoid plus cyclophosphamide; LNSCn, zero to low-dose glucocorticoid plus calcineurin inhibitors; SE, glucocorticoid alone; SP, supportive care alone.

Treatment	SUCRA	PrBest	Mean rank
HMSCh	13.7	0.1	6.2
HMSCn	96.6	83.3	1.2
HMSCt	78.4	2.7	2.3
LNSCn	51.7	0.5	3.9
LNSCt	61.9	13	3.3
SE	24.8	0.3	5.5
SP	22.9	0	5.6

Relapse Rate

A total of 10 studies involving 96 relapsed in 484 patients during the follow-up. Seven therapeutic regimens were involved: HMSCh (one trial, 11 patients with relapse), HMSCn (three trials, 10 patients with relapse), HMSCt (eight trials, 36 patients with relapse), LNSCn (two trials, 12 patients with relapse), R (one trial, three patients with relapse), SE (three trials, 15 patients with relapse), SP (two trials, nine patients with relapse). The network map is demonstrated in supplementary figure S2A. No significant difference was found among the therapeutic regimens by network meta-analysis (supplementary table S10). Details of SUCRA ranking are demonstrated in supplementary table S11 and figure S2B.

Discussion

In our network meta-analysis on treatments of IMN, it is found that regimens of moderate- to high-dose glucocorticoids plus either chlorambucil, cyclophosphamide, or CNIs increased the probability of remission compared to solely supportive care. Moreover, the regimen of zero to low-dose glucocorticoids plus CNIs and the regimen of rituximab alone also showed more effectiveness than supportive care regarding remission. Moderate- to high-dose glucocorticoids plus CNIs showed the highest probability of total remission and reduction of proteinuria. Moderate- to high-dose glucocorticoids were not demonstrated to increase the infection rate compared with zero to low-dose when added to either cyclophosphamide or CNIs.

Glucocorticoids have been widely administrated in glomerular diseases. Glucocorticoids exert anti-inflammatory and immunosuppressive functions through genomic and nongenomic effects [18]. The dosage of glucocorticoids varies in different research on IMN. Large-dose impulsion sequential by a medium dose of glucocorticoids plus immunosuppressants was applied in several studies [19,20]. Moreover, an initial high dose of glucocorticoid plus immunosuppressants has also been mentioned [21,22]. Besides, a regimen of low-dose or even no glucocorticoids with immunosuppressants especially CNIs has shown effectiveness in remission of IMN [23,24]. Seldom studies have compared the dosage of glucocorticoids directly and the optimal dosage of glucocorticoids hasn’t been recommended in 2021 KDIGO guideline [5]. In our study, different dosages of glucocorticoids with the same immunosuppressants (cyclophosphamide or CNIs) were compared through network meta-analysis. Regimens of CNIs regardless of the dosage of glucocorticoids had a higher probability of total remission compared with supportive care alone, while CNIs plus moderate- to high-dose glucocorticoids had the highest probability. Concerning reduction of proteinuria, moderate- to high-dose glucocorticoids showed superiority to zero to low-dose when accompanied with CNIs. It is naturally understood that the higher the dosage of the glucocorticoid, the more the effectiveness of disease remission.

Side effects of glucocorticoids are usually dose- and time-dependent [18]. It is reported that the side effects of the administration of glucocorticoids plus cyclosporine were significantly higher than those of cyclosporine alone [24]. In our study, we focused on the adverse effects of infection. However, we did not find that moderate- to high-dose glucocorticoids showed a higher probability of infection rate compared with zero to low-dose when added to either cyclophosphamide or CNIs. The result might be due to the rapid reduction of protein leakage after administration of adequate glucocorticoids or the quick tapering of glucocorticoids after remission, which might reduce the risk of infection, in the regimens of moderate- to high-dose glucocorticoids plus cyclophosphamide or CNIs.

CNIs, such as cyclosporine and tacrolimus, exert their immunosuppressive effect by indirectly affecting B-cell function. It has been widely proven from clinical studies that CNIs-based management increased the probability of PR and CR in IMN patients [6]. It was reported that the reduction of proteinuria was more significant in the CNIs group compared with the cyclophosphamide group [6,25]. Our network meta-analysis demonstrated that moderate- to high-dose glucocorticoids combined with CNIs seemed to show the highest probability of total remission and be the best regimen in reducing 24-hour UTP, which was consistent with the previous studies. The superiority of CNIs in the reduction of proteinuria may be owing to the direct target on the podocyte [26].

Some limitations should be taken into account when interpreting our results. First, the outcome of renal function after follow-up has not been investigated due to the insufficiency of evidence for some regimens. Secondly, the serum level of anti-PLA2R antibody has not been estimated because only a few studies published recently had mentioned this specific biomarker of IMN. Thirdly, the sample sizes of some studies were only pretty limited, which reduced the level of evidence in our study. Hence, more new studies need to be included in the future.

## Conclusions

In conclusion, moderate- to high-dose glucocorticoids showed superiority in proteinuria remission compared with zero to low-dose glucocorticoids, when combined with CNIs in IMN, with no increasing risk of infection.

## Figures and Tables

**Figure 5:**
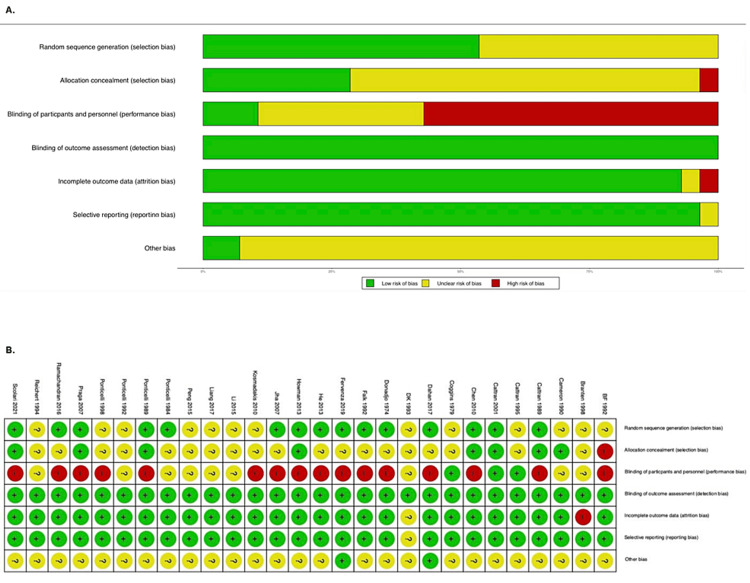
Supplementary figure 1. Risk-of-bias assessments of the included studies. "+", low risk of bias; "?", unclear risk of bias; "-", high risk of bias.

**Figure 6:**
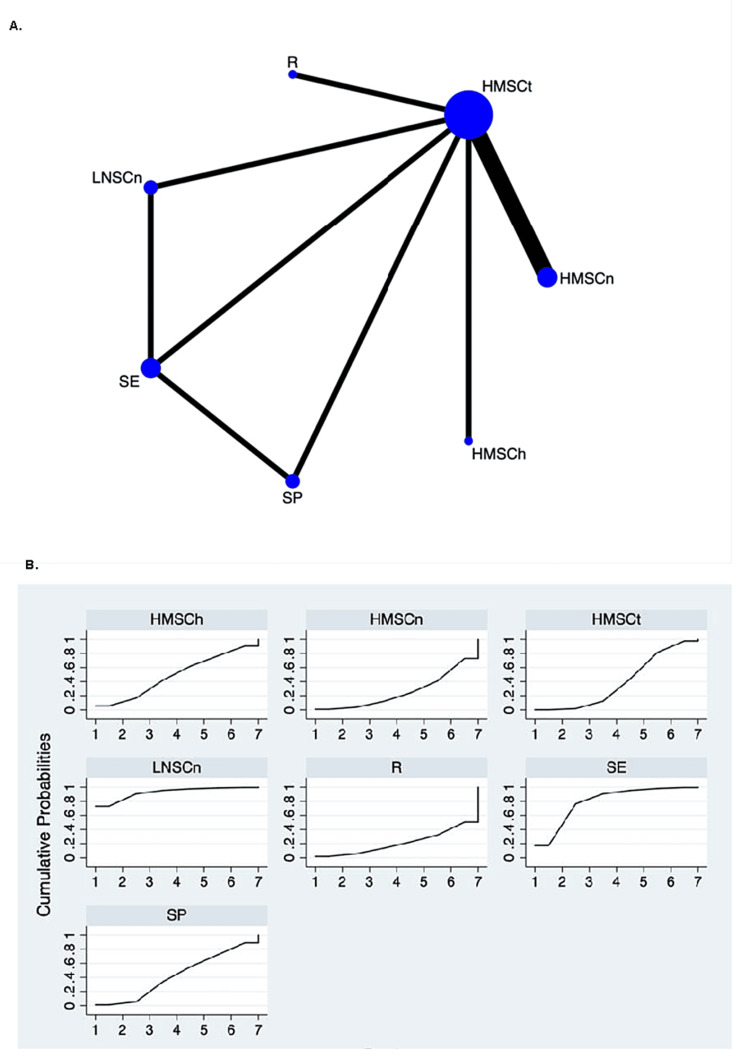
Supplementary figure 2. Network map and SURCA plot for relapse rate in IMN. (A) Network map. Nodes indicate the treatment regimens that are evaluated in the study. The size of the node is proportional to the number of trials evaluating the treatment. Lines represent head-to-head comparisons of the two treatment regimens indicated by the connected nodes. The thickness of lines is weighted according to the number of trials comparing the two connected treatment regimens. (B) SURCA plot. IMN, idiopathic membranous nephropathy; HMSCh, moderate- to high-dose glucocorticoid plus chlorambucil; HMSCn, moderate- to high-dose glucocorticoid plus calcineurin inhibitors; HMSCt, moderate- to high-dose glucocorticoid plus cyclophosphamide; LNSCn, zero to low-dose glucocorticoid plus calcineurin inhibitors; R, rituximab alone; SE, glucocorticoid alone; SP, supportive care alone.

## Appendices

Supplementary references

s1.   Jha V, Ganguli A, Saha TK, et al. A randomized, controlled trial of steroids and cyclophosphamide in adults with nephrotic syndrome caused by idiopathic membranous nephropathy. J Am Soc Nephrol. Jun 2007;18(6):1899-904. 10.1681/asn.2007020166

s2.   Ponticelli C, Altieri P, Scolari F, et al. A randomized study comparing methylprednisolone plus chlorambucil versus methylprednisolone plus cyclophosphamide in idiopathic membranous nephropathy. J Am Soc Nephrol. Mar 1998;9(3):444-50. 10.1681/asn.V93444

s3.   Ponticelli C, Zucchelli P, Passerini P, et al. A randomized trial of methylprednisolone and chlorambucil in idiopathic membranous nephropathy. N Engl J Med. Jan 5 1989;320(1):8-13. 10.1056/nejm198901053200102

s4.   He L, Peng Y, Liu H, et al. Treatment of idiopathic membranous nephropathy with combination of low-dose tacrolimus and corticosteroids. J Nephrol. May-Jun 2013;26(3):564-71. 10.5301/jn.5000199

s5.   Ramachandran R, Hn HK, Kumar V, et al. Tacrolimus combined with corticosteroids versus Modified Ponticelli regimen in treatment of idiopathic membranous nephropathy: Randomized control trial. Nephrology (Carlton). Feb 2016;21(2):139-46. 10.1111/nep.12569

s6.   Liang Q, Li H, Xie X, Qu F, Li X, Chen J. The efficacy and safety of tacrolimus monotherapy in adult-onset nephrotic syndrome caused by idiopathic membranous nephropathy. Ren Fail. Nov 2017;39(1):512-518. 10.1080/0886022x.2017.1325371

s7.   Peng L, Wei SY, Li LT, He YX, Li B. Comparison of different therapies in high-risk patients with idiopathic membranous nephropathy. J Formos Med Assoc. Jan 2016;115(1):11-8. 10.1016/j.jfma.2015.07.021

s8.   Ponticelli C, Zucchelli P, Passerini P, Cesana B. Methylprednisolone plus chlorambucil as compared with methylprednisolone alone for the treatment of idiopathic membranous nephropathy. The Italian Idiopathic Membranous Nephropathy Treatment Study Group. N Engl J Med. Aug 27 1992;327(9):599-603. 10.1056/nejm199208273270904

s9.   Cattran DC, Appel GB, Hebert LA, et al. Cyclosporine in patients with steroid-resistant membranous nephropathy: a randomized trial. Kidney Int. Apr 2001;59(4):1484-90. 10.1046/j.1523-1755.2001.0590041484.x

s10. Chen M, Li H, Li XY, et al. Tacrolimus combined with corticosteroids in treatment of nephrotic idiopathic membranous nephropathy: a multicenter randomized controlled trial. Am J Med Sci. Mar 2010;339(3):233-8. 10.1097/MAJ.0b013e3181ca3a7d

s11. Cattran DC, Delmore T, Roscoe J, et al. A randomized controlled trial of prednisone in patients with idiopathic membranous nephropathy. N Engl J Med. Jan 26 1989;320(4):210-5. 10.1056/nejm198901263200403

s12. Reichert LJ, Huysmans FT, Assmann K, Koene RA, Wetzels JF. Preserving renal function in patients with membranous nephropathy: daily oral chlorambucil compared with intermittent monthly pulses of cyclophosphamide. Ann Intern Med. Sep 1 1994;121(5):328-33. 10.7326/0003-4819-121-5-199409010-00003

s13. Li MX, Yu YW, Zhang ZY, Zhao HD, Xiao FL. Administration of low-dose cyclosporine alone for the treatment of elderly patients with membranous nephropathy. Genet Mol Res. Mar 30 2015;14(1):2665-73. 10.4238/2015.March.30.27

s14. Kosmadakis G, Filiopoulos V, Smirloglou D, Skarlas P, Georgoulias C, Michail S. Comparison of immunosuppressive therapeutic regimens in patients with nephrotic syndrome due to idiopathic membranous nephropathy. Ren Fail. Jun 2010;32(5):566-71. 10.3109/08860221003728754

s15. Branten AJ, Reichert LJ, Koene RA, Wetzels JF. Oral cyclophosphamide versus chlorambucil in the treatment of patients with membranous nephropathy and renal insufficiency. QJM. May 1998;91(5):359-66. 10.1093/qjmed/91.5.359

s16. A controlled study of short-term prednisone treatment in adults with membranous nephropathy. N Engl J Med. Dec 13 1979;301(24):1301-6. 10.1056/nejm197912133012401

s17. Fervenza FC, Appel GB, Barbour SJ, et al. Rituximab or cyclosporine in the treatment of membranous nephropathy. N Engl J Med. Jul 4 2019;381(1):36-46. 10.1056/NEJMoa1814427

s18. Dahan K, Debiec H, Plaisier E, et al. Rituximab for severe membranous nephropathy: A 6-month trial with extended follow-up. J Am Soc Nephrol. Jan 2017;28(1):348-358. 10.1681/asn.2016040449

s19. Praga M, Barrio V, Juárez GF, Luño J. Tacrolimus monotherapy in membranous nephropathy: A randomized controlled trial. Kidney Int. May 2007;71(9):924-30. 10.1038/sj.ki.5002215

s20. Murphy BF, McDonald I, Fairley KF, Kincaid-Smith PS. Randomized controlled trial of cyclophosphamide, warfarin and dipyridamole in idiopathic membranous glomerulonephritis. Clin Nephrol. May 1992;37(5):229-34. https://pubmed.ncbi.nlm.nih.gov/1606772/.

s21. Cattran DC, Greenwood C, Ritchie S, et al. A controlled trial of cyclosporine in patients with progressive membranous nephropathy. Canadian Glomerulonephritis Study Group. Kidney Int. Apr 1995;47(4):1130-5. 10.1038/ki.1995.161

s22. Pahari DK, Das S, Dutta BN, Banerjee D. Prognosis and management of membraneous nephropathy. J Assoc Physicians India. Jun 1993;41(6):350-1. https://pubmed.ncbi.nlm.nih.gov/8005970/.

s23. Donadio JV, Jr., Holley KE, Anderson CF, Taylor WF. Controlled trial of cyclophosphamide in idiopathic membranous nephropathy. Kidney Int. Dec 1974;6(6):431-9. 10.1038/ki.1974.129

s24. Scolari F, Delbarba E, Santoro D, et al. Rituximab or cyclophosphamide in the treatment of membranous nephropathy: The RI-CYCLO randomized trial. J Am Soc Nephrol. Mar 1 2021;32(4):972-82. 10.1681/asn.2020071091

s25. Ponticelli C, Zucchelli P, Imbasciati E, et al. Controlled trial of methylprednisolone and chlorambucil in idiopathic membranous nephropathy. N Engl J Med. Apr 12 1984;310(15):946-50. 10.1056/nejm198404123101503

s26. Howman A, Chapman TL, Langdon MM, et al. Immunosuppression for progressive membranous nephropathy: A UK randomised controlled trial. Lancet. Mar 2 2013;381(9868):744-51. 10.1016/s0140-6736(12)61566-9

s27. Falk RJ, Hogan SL, Muller KE, Jennette JC. Treatment of progressive membranous glomerulopathy. A randomized trial comparing cyclophosphamide and corticosteroids with corticosteroids alone. The Glomerular Disease Collaborative Network. Ann Intern Med. Mar 15 1992;116(6):438-45. 10.7326/0003-4819-116-6-438

s28. Cameron JS, Healy MJ, Adu D. The Medical Research Council trial of short-term high-dose alternate day prednisolone in idiopathic membranous nephropathy with nephrotic syndrome in adults. The MRC Glomerulonephritis Working Party. Q J Med. Feb 1990;74(274):133-56. https://pubmed.ncbi.nlm.nih.gov/2189149/.

**Table 8:** Supplementary table S1. Search strategy

Database	PubMed
URL	https://www.ncbi.nlm.nih.gov/pubmed/
Search strategy	#1 ((((((((((((((((((((((Glomerulonephritis, Membranous[MeSH Terms]) OR Glomerulonephritides, Membranous[Title/Abstract]) OR Membranous Glomerulonephritides[Title/Abstract]) OR Membranous Glomerulonephritis[Title/Abstract]) OR Nephropathy, Membranous[Title/Abstract]) OR Membranous Glomerulopathy[Title/Abstract]) OR Glomerulopathy, Membranous[Title/Abstract]) OR Membranous Nephropathy[Title/Abstract]) OR Extramembranous Glomerulopathy[Title/Abstract]) OR Glomerulopathy, Extramembranous[Title/Abstract]) OR Membranous Glomerulonephropathy[Title/Abstract]) OR Glomerulonephropathy, Membranous[Title/Abstract]) OR Idiopathic Membranous Glomerulonephritis[Title/Abstract]) OR Glomerulonephritides, Idiopathic Membranous[Title/Abstract]) OR Glomerulonephritis, Idiopathic Membranous[Title/Abstract]) OR Idiopathic Membranous Glomerulonephritides[Title/Abstract]) OR Membranous Glomerulonephritides, Idiopathic[Title/Abstract]) OR Membranous Glomerulonephritis, Idiopathic[Title/Abstract]) OR Idiopathic Membranous Nephropathy[Title/Abstract]) OR Membranous Nephropathy, Idiopathic[Title/Abstract]) OR Nephropathy, Idiopathic Membranous[Title/Abstract]) OR Heymann Nephritis[Title/Abstract]) OR Nephritis, Heymann[Title/Abstract] #2 ((((Randomized Controlled Trial[Publication Type]) OR Controlled Clinical Trial[Publication Type]) OR Clinical Trials[Title/Abstract]) OR Randomized[Title/Abstract]) OR drug therapy[Title/Abstract] #3 ((meta analysis[Title/Abstract]) OR cochrane review[Title/Abstract]) OR systematic review[Title/Abstract] #4 (#1) AND #2 #5 (#1) AND #3 #6 (#4) OR #5
Result	297
Database	Cochrane library
URL	http://www.cochranelibrary.com/
Search strategy	#1 Glomerulonephritis, Membranous:ti,ab,kw or Glomerulonephritides, Membranous:ti,ab,kw or Membranous Glomerulonephritides:ti,ab,kw or Membranous Glomerulonephritis:ti,ab,kw or Nephropathy, Membranous:ti,ab,kw (Word variations have been searched) 234 #2 Membranous Glomerulopathy:ti,ab,kw or Glomerulopathy, Membranous:ti,ab,kw or Membranous Nephropathy:ti,ab,kw or Extramembranous Glomerulopathy:ti,ab,kw or Glomerulopathy, Extramembranous:ti,ab,kw (Word variations have been searched) 195 #3 "membranous glomerulonephropathy":ti,ab,kw or Glomerulonephropathy, Membranous:ti,ab,kw or "idiopathic membranous glomerulonephritis":ti,ab,kw or Glomerulonephritides, Idiopathic Membranous:ti,ab,kw or Glomerulonephritis, Idiopathic Membranous:ti,ab,kw (Word variations have been searched) 113 #4 "idiopathic membranous glomerulonephritides":ti,ab,kw or Membranous Glomerulonephritides, Idiopathic:ti,ab,kw or Membranous Glomerulonephritis, Idiopathic:ti,ab,kw or “Idiopathic Membranous Nephropathy”:ti,ab,kw or Membranous Nephropathy, Idiopathic:ti,ab,kw (Word variations have been searched) 122 #5 Nephropathy, Idiopathic Membranous:ti,ab,kw or "Heymann nephritis":ti,ab,kw or Nephritis, Heymann:ti,ab,kw (Word variations have been searched) 110 #6 #1 or #2 or #3 or #4 or #5
Result	417
Database	EMbase
URL	https://www.embase.com/
Search strategy	('membranous glomerulonephritis'/exp OR 'membranous glomerulonephritis' OR 'membranous glomerulonephritides':ti,ab,kw OR 'membranous glomerulopathy':ti,ab,kw OR 'membranous nephropathy':ti,ab,kw OR 'extramembranous glomerulopathy':ti,ab,kw OR 'membranous glomerulonephropathy':ti,ab,kw OR 'idiopathic membranous glomerulonephritis':ti,ab,kw OR 'idiopathic membranous glomerulonephritides':ti,ab,kw OR 'idiopathic membranous nephropathy':ti,ab,kw OR 'heymann nephritis':ti,ab,kw) AND ([cochrane review]/lim OR [systematic review]/lim OR [meta analysis]/lim OR [controlled clinical trial]/lim OR [randomized controlled trial]/lim)
Result	408
Database	Clinical trials
URL	https://clinicaltrials.gov/
Search strategy	Condition or disease:" Membranous Glomerulonephritides " OR " Membranous Glomerulonephritis " OR " Membranous Glomerulopathy " OR " Membranous Nephropathy " OR " Extramembranous Glomerulopathy" OR " Membranous Glomerulonephropathy " OR ”Idiopathic Membranous Glomerulonephritis" OR "Idiopathic Membranous Glomerulonephritides" OR "Idiopathic Membranous Nephropathy" OR "Heymann Nephritis" Study type: Interventional studies(clinical trials) Study Results: studies with results
Result	7

**Table 9:** Supplementary table S2. Evaluation of local inconsistency for total remission rate with node-split model. The ratio of risk ratios (RRs) for direct and indirect estimates with their 95% confidence intervals (CIs). A CI including 1 indicates there is no statistically detectable inconsistency between direct and indirect treatment estimates. Bold values denote statistical significance. HMSCh, moderate- to high-dose glucocorticoid plus chlorambucil; HMSCn, moderate- to high-dose glucocorticoid plus calcineurin inhibitors; HMSCt, moderate- to high-dose glucocorticoid plus cyclophosphamide; LNSCn, zero to low-dose glucocorticoid plus calcineurin inhibitors; LNSCt, zero to low-dose glucocorticoid plus cyclophosphamide; R, rituximab alone; SE, glucocorticoid alone; SP, supportive care alone.

Comparison	Direct risk ratio	Indirect risk ratio	Network risk ratio	p-Value
HMSCt vs HMSCh	1.500 (0.720,3.200)	0.900 (0.380,2.200)	1.200 (0.700,2.100)	0.335
SE vs HMSCh	0.730 (0.220,2.400)	0.650 (0.270,1.500)	0.680 (0.350,1.300)	0.867
SP vs HMSCh	0.410 (0.170,0.930)	0.760 (0.340,1.700)	0.560 (0.320,1.000)	0.251
HMSCt vs HMSCn	0.850 (0.470,1.500)	0.940 (0.210,3.900)	0.860 (0.510,1.400)	0.892
LNSCn vs HMSCn	0.930 (0.250,3.500)	0.840 (0.370,2.000)	0.860 (0.450,1.700)	0.892
LNSCn vs HMSCt	0.820 (0.460,1.400)	1.200 (0.730,2.100)	1.000 (0.600,1.800)	0.268
R vs HMSCt	0.850 (0.260,2.800)	1.100 (0.390,2.900)	0.960 (0.470,2.000)	0.762
SE vs HMSCt	0.680 (0.210,2.200)	0.510 (0.230,1.100)	0.560 (0.300,1.000)	0.656
SP vs HMSCt	0.830 (0.430,1.800)	0.330 (0.180,0.560)	0.470 (0.270,0.770)	0.031
R vs LNSCn	1.100 (0.360,3.800)	0.830 (0.290,2.200)	0.960 (0.460,1.900)	0.641
SE vs LNSCn	0.270 (0.068,0.970)	0.720 (0.320,1.500)	0.560 (0.280,1.100)	0.189
SP vs LNSCn	0.620 (0.250,1.400)	0.360 (0.150,0.810)	0.470 (0.250,0.800)	0.333
SP vs R	0.520 (0.150,1.800)	0.460 (0.170,1.200)	0.480 (0.230,1.000)	0.874
SP vs SE	0.740 (0.300,1.800)	0.930 (0.390,2.200)	0.830 (0.460,1.500)	0.687

**Table 10:** Supplementary table S3. Evaluation of local inconsistency for adverse event (infection) rate with node-split model. The ratio of risk ratios (RRs) for direct and indirect estimates with their 95% confidence intervals (CIs). A CI including 1 indicates there is no statistically detectable inconsistency between direct and indirect treatment estimates. Bold values denote statistical significance. HMSCh, moderate- to high-dose glucocorticoid plus chlorambucil; HMSCn, moderate- to high-dose glucocorticoid plus calcineurin inhibitors; HMSCt, moderate- to high-dose glucocorticoid plus cyclophosphamide; LNSCn, zero to low-dose glucocorticoid plus calcineurin inhibitors; LNSCt, zero to low-dose glucocorticoid plus cyclophosphamide; R, rituximab alone; SE, glucocorticoid alone; SP, supportive care alone.

Comparison	Direct risk ratio	Indirect risk ratio	Network risk ratio	p-Value
HMSCt vs HMSCh	0.130 (0.007,0.830)	2.200 (0.100,41.000)	0.320 (0.038,1.500)	0.096
LNSCn vs HMSCh	2.700 (0.210,39.000)	0.036 (0.001,0.250)	0.160 (0.012,1.000)	0.007
SP vs HMSCh	0.350 (0.017,3.600)	0.170 (0.005,1.200)	0.250 (0.020,1.600)	0.648
HMSCt vs HMSCn	0.770 (0.130,4.000)	0.750 (0.006,79.000)	0.780 (0.170,3.400)	0.996
LNSCn vs HMSCn	0.360 (0.004,20.000)	0.380 (0.022,4.100)	0.400 (0.041,2.800)	0.989
LNSCn vs HMSCt	0.052 (0.001,0.890)	1.300 (0.190,8.600)	0.510 (0.078,2.500)	0.062
R vs HMSCt	0.380 (0.013,11.000)	0.520 (0.019,11.000)	0.450 (0.049,3.800)	0.882
SP vs HMSCt	0.680 (0.026,9.600)	0.860 (0.072,12.000)	0.790 (0.120,4.500)	0.891
R vs LNSCn	0.820 (0.030,22.000)	1.000 (0.047,30.000)	0.880 (0.110,8.500)	0.919
SP vs LNSCn	1.100 (0.160,13.000)	3.200 (0.140,64.000)	1.500 (0.280,9.700)	0.544
SP vs R	0.960 (0.008,1.1e+02)	2.100 (0.120,38.000)	1.700 (0.170,17.000)	0.758

**Table 11:** Supplementary table S4. Evaluation of local inconsistency for change in 24-hour UTP with node-split model. The mean difference (MDs) for direct and indirect estimates with their 95% confidence intervals (CIs). A CI including 0 indicates there is no statistically detectable inconsistency between direct and indirect treatment estimates. Bold values denote statistical significance. 24-hour UTP, 24-hour urine total protein; HMSCh, moderate- to high-dose glucocorticoid plus chlorambucil; HMSCn, moderate- to high-dose glucocorticoid plus calcineurin inhibitors; HMSCt, moderate- to high-dose glucocorticoid plus cyclophosphamide; LNSCn, zero to low-dose glucocorticoid plus calcineurin inhibitors; LNSCt, zero to low-dose glucocorticoid plus cyclophosphamide; SE, glucocorticoid alone; SP, supportive care alone.

Comparison	Direct mean difference	Indirect mean difference	Network mean difference	p-Value
HMSCt vs HMSCh	-6.700 (-10.000,-3.400)	-0.380 (-3.700,3.200)	-3.800 (-6.800,-1.100)	0.011
SE vs HMSCh	1.100 (-1.300,3.500)	-5.200 (-9.300,-1.200)	-0.610 (-3.400,1.800)	0.011
LNSCn vs HMSCt	1.800 (0.010,3.500)	3.100 (-4.900,11.000)	1.800 (-0.012,3.600)	0.731
SE vs HMSCt	-0.810 (-6.900,5.400)	3.900 (1.200,7.400)	3.200 (0.520,5.800)	0.165
SP vs HMSCt	2.800 (-0.510,6.100)	5.300 (0.330,9.200)	3.100 (1.300,5.400)	0.298
SP vs LNSCn	1.100 (-1.500,4.500)	4.000 (-4.100,9.900)	1.300 (-0.490,3.600)	0.393
SP vs SE	0.910 (-1.900,3.700)	-2.200 (-5.900,2.700)	-0.028 (-2.400,2.700)	0.211

**Table 12:** Supplementary table S5. Evaluation of local inconsistency for relapse rate with node-split model. The ratio of risk ratios (RRs) for direct and indirect estimates with their 95% confidence intervals (CIs). A CI including 1 indicates there is no statistically detectable inconsistency between direct and indirect treatment estimates. HMSCn, moderate- to high-dose glucocorticoid plus calcineurin inhibitors; HMSCt, moderate- to high-dose glucocorticoid plus cyclophosphamide; LNSCn, zero to low-dose glucocorticoid plus calcineurin inhibitors; SE, glucocorticoid alone; SP, supportive care alone.

Comparison	Direct risk ratio	Indirect risk ratio	Network risk ratio	p-Value
LNSCn vs HMSCt	5.6e+05 (3.300,6.9e+19)	3.300 (0.230,63.000)	6.300 (0.950,1.2e+02)	0.07
SE vs HMSCt	2.400 (0.170,41.000)	4.200 (0.470,1.1e+02)	3.100 (0.690,20.000)	0.723
SP vs HMSCt	1.000 (0.100,9.100)	1.600 (0.084,55.000)	1.100 (0.240,6.200)	0.75
SE vs LNSCn	0.760 (0.069,5.200)	5.8e-07 (1.2e-19,0.770)	0.500 (0.043,2.600)	0.064
SP vs SE	0.410 (0.044,3.500)	0.230 (0.006,4.900)	0.370 (0.066,1.600)	0.736

**Table 13:** Supplementary table S6. Outcomes of conventional pairwise meta-analysis for total remission rate. HMSCh, moderate- to high-dose glucocorticoid plus chlorambucil; HMSCn, moderate- to high-dose glucocorticoid plus calcineurin inhibitors; HMSCt, moderate- to high-dose glucocorticoid plus cyclophosphamide; LNSCn, zero to low-dose glucocorticoid plus calcineurin inhibitors; LNSCt, zero to low-dose glucocorticoid plus cyclophosphamide; R, rituximab alone; SE, glucocorticoid alone; SP, supportive care alone; NA, no available. Tau^2^ represents between-study heterogeneity characterized by standard deviation. Bold values denote statistical significance.

Comparisons	Number of trials	Number of patients	Tau^2^	I^2^	RR (95%CI)
HMSCh vs HMSCt	3	137	0.822	77%	0.69 (0.22,2.20)
HMSCh vs SE	1	92	NA	NA	1.37 (1.01,1.85)
HMSCh vs SP	2	143	0	0%	2.42 (1.68,3.48)
HMSCn vs HMSCt	4	259	0.011	35%	1.17 (0.98,1.39)
LNSCn vs HMSCn	1	27	NA	NA	0.93 (0.50,1.73)
LNSCt vs SP	2	62	0	0%	2.32 (1.01,5.32)
HMSCt vs LNSCn	2	76	0.065	64%	1.23 (0.80,1.90)
R vs HMSCt	1	74	NA	NA	0.85 (0.62,1.17)
HMSCt vs SE	1	71	NA	NA	1.46 (1.11,1.92)
HMSCt vs SP	2	111	0.238	89%	1.41 (0.69,2.88)
R vs LNSCn	1	130	NA	NA	1.15 (0.85,1.56)
LNSCn vs SE	1	51	NA	NA	3.45 (1.54,7.71)
LNSCn vs SP	3	85	1.423	87%	2.06 (0.44,9.71)
R vs SP	1	75	NA	NA	1.90 (1.15,3.13)
SE vs SP	2	230	0.442	84%	1.34 (0.49,3.68)

**Table 14:** Supplementary table S7. Outcomes of conventional pairwise meta-analysis for adverse event (infection) rate. HMSCh, moderate- to high-dose glucocorticoid plus chlorambucil; HMSCn, moderate- to high-dose glucocorticoid plus calcineurin inhibitors; HMSCt, moderate- to high-dose glucocorticoid plus cyclophosphamide; LNSCn, zero to low-dose glucocorticoid plus calcineurin inhibitors; R, rituximab alone; SE, glucocorticoid alone; SP, supportive care alone; NA, no available. Bold values denote statistical significance. Tau^2^ represents between-study heterogeneity characterized by standard deviation.

Comparisons	Number of trials	Number of patients	Tau^2^	I^2^	RR (95%CI)
HMSCh vs HMSCt	3	137	0.729	32%	3.43 (0.79,14.94)
HMSCh vs LNSCn	1	69	NA	NA	0.41 (0.12,1.41)
HMSCh vs SE	1	92	NA	NA	5.22 (0.26,105.77)
HMSCh vs SP	2	151	0	0%	1.89 (0.41,8.60)
HMSCn vs HMSCt	4	259	0.2	42%	1.10 (0.56,2.19)
HMSCt vs LNSCn	2	76	0	0%	7.23 (1.35,38.71)
HMSCt vs R	1	74	NA	NA	2.50 (1.09,5.73)
HMSCt vs SP	2	111	0.094	7%	0.80 (0.33,1.94)
LNSCn vs HMSCn	1	27	NA	NA	0.46 (0.05,4.53)
LNSCn vs SP	4	158	1.814	61%	0.91 (0.13,6.53)
LNSCt vs SP	1	22	NA	NA	NA
R vs LNSCn	1	130	NA	NA	0.82 (0.49,1.38)
R vs SP	1	75	NA	NA	1.03 (0.07,15.82)

**Table 15:** Supplementary table S8. Outcomes of conventional pairwise meta-analysis for change in 24-hour UTP. 24-hour UTP, 24-hour urine total protein; HMSCh, moderate- to high-dose glucocorticoid plus chlorambucil; HMSCn, moderate- to high-dose glucocorticoid plus calcineurin inhibitors; HMSCt, moderate- to high-dose glucocorticoid plus cyclophosphamide; LNSCn, zero to low-dose glucocorticoid plus calcineurin inhibitors; LNSCt, zero to low-dose glucocorticoid plus cyclophosphamide; SE, glucocorticoid alone; SP, supportive care alone; NA, no available. Tau^2^ represents between-study heterogeneity characterized by standard deviation. Bold values denote statistical significance.

Comparisons	Number of trials	Number of patients	Tau^2^		I^2^	MD (95%CI)
HMSCh vs SE	1	50	NA		NA	-1.10 (-3.13,0.93)
HMSCn vs HMSCt	4	259	0		0%	-1.19 (-1.82,-0.55)
HMSCt vs HMSCh	1	32	NA		NA	-6.70 (-9.62,-3.78)
HMSCt vs LNSCn	2	76	0		0%	-1.78 (-2.38,-1.18)
HMSCt vs SE	1	26	NA		NA	0.80 (-4.94,6.54)
HMSCt vs SP	1	18	NA		NA	-2.80 (-3.41,-2.19)
LNSCn vs SP	2	37	0		0%	-1.03 (-1.72,-0.34)
LNSCt vs SP	1	22	NA		NA	-2.10 (-4.90,0.70)
SE vs SP	1	103	NA		NA	-0.90 (-2.91,1.11)

**Table 16:** Supplementary table S9. Outcomes of conventional pairwise meta-analysis for relapse rate. HMSCh, moderate- to high-dose glucocorticoid plus chlorambucil; HMSCn, moderate- to high-dose glucocorticoid plus calcineurin inhibitors; HMSCt, moderate- to high-dose glucocorticoid plus cyclophosphamide; LNSCn, zero to low-dose glucocorticoid plus calcineurin inhibitors; R, rituximab alone; SE, glucocorticoid alone; SP, supportive care alone; NA, no available. Tau^2^ represents between-study heterogeneity characterized by standard deviation. Bold values denote statistical significance.

Comparisons	Number of trials	Number of patients	Tau^2^	I^2^	RR (95%CI)
HMSCh vs HMSCt	1	76	NA	NA	1.22 (0.59,2.53)
HMSCn vs HMSCt	3	145	0	0%	0.71 (0.31,1.62)
HMSCt vs LNSCn	1	47	NA	NA	0.15 (0.01,2.73)
R vs HMSCt	1	50	NA	NA	0.59 (0.16,2.09)
HMSCt vs SE	1	55	NA	NA	0.44 (0.08,2.45)
HMSCt vs SP	1	50	NA	NA	0.94 (0.33,2.67)
LNSCn vs SE	1	26	NA	NA	1.07 (0.33,3.49)
SE vs SP	1	35	NA	NA	2.38 (1.02,5.52)

**Table 17:** Supplementary table S10. The league table of all comparisons of relapse rate. The league table of all comparisons of relapse rate. The summary estimates are risk ratios (RRs) and 95% confidence intervals. The column-defining treatment is compared to the row-defining treatment, and RRs >1 favor the column-defining treatment. HMSCh, moderate- to high-dose glucocorticoid plus chlorambucil; HMSCn, moderate/high-dose glucocorticoid plus calcineurin inhibitors; HMSCt, moderate- to high-dose glucocorticoid plus cyclophosphamide; LNSCn, zero to low-dose glucocorticoid plus calcineurin inhibitors; R, rituximab alone; SE, glucocorticoid alone; SP, supportive care alone.

HMSCh						
1.7 (0.18, 14.87)	HMSCn					
1.23 (0.2, 7.52)	0.72 (0.21, 2.73)	HMSCt				
0.2 (0.01, 2.37)	0.12 (0, 1.11)	0.16 (0.01, 1.09)	LNSCn			
2.22 (0.15, 38)	1.3 (0.12, 17.24)	1.77 (0.24, 17.06)	11.15 (0.76, 478.46)	R		
0.41 (0.03, 3.84)	0.24 (0.02, 1.65)	0.33 (0.05, 1.45)	1.94 (0.39, 22.13)	0.18 (0.01, 2.1)	SE	
1.11 (0.09, 11.33)	0.65 (0.08, 4.83)	0.9 (0.17, 4.08)	5.41 (0.75, 100.33)	0.5 (0.03, 6.06)	2.69 (0.65, 14.9)	SP

**Table 18:** Supplementary table S11. SUCRA value of relapse rate. HMSCh, moderate- to high-dose glucocorticoid plus chlorambucil; HMSCn, moderate/high-dose glucocorticoid plus calcineurin inhibitors; HMSCt, moderate- to high-dose glucocorticoid plus cyclophosphamide; LNSCn, zero to low-dose glucocorticoid plus calcineurin inhibitors; R, rituximab alone; SE, glucocorticoid alone; SP, supportive care alone.

Treatment	SUCRA	PrBest	Mean rank
HMSCh	48.9	5.6	4.1
HMSCn	25.7	0.9	5.5
HMSCt	39.4	0.2	4.6
LNSCn	92.6	73.1	1.4
R	21	1.8	5.7
SE	79.6	17.2	2.2
SP	42.9	1.2	4.4
